# Are We Ready to Recommend Capsaicin for Disorders Other Than Neuropathic Pain?

**DOI:** 10.3390/nu15204469

**Published:** 2023-10-21

**Authors:** Janayne L. Silva, Elandia A. Santos, Jacqueline I. Alvarez-Leite

**Affiliations:** Departamento de Bioquímica e Imunologia, Universidade Federal de Minas Gerais, Belo Horizonte 30161-970, MG, Brazil; janayneluihan@gmail.com (J.L.S.); elandianutri@gmail.com (E.A.S.)

**Keywords:** capsaicin, pepper, diabetes, obesity, inflammation, gut microbiota

## Abstract

Capsaicin, a lipophilic, volatile compound, is responsible for the pungent properties of chili peppers. In recent years, a significant increase in investigations into its properties has allowed the production of new formulations and the development of tools with biotechnological, diagnostic, and potential therapeutic applications. Most of these studies show beneficial effects, improving antioxidant and anti-inflammatory status, inducing thermogenesis, and reducing white adipose tissue. Other mechanisms, including reducing food intake and improving intestinal dysbiosis, are also described. In this way, the possible clinical application of such compound is expanding every year. This opinion article aims to provide a synthesis of recent findings regarding the mechanisms by which capsaicin participates in the control of non-communicable diseases such as obesity, diabetes, and dyslipidemia.

## 1. Introduction

Capsaicinoids are a group of lipophilic and volatile compounds with different pungency intensities characterized by a vanilloid ring. They have diverse biological activities and applications, from food flavoring to therapeutic adjuncts [1]. Among these, capsaicin (trans-8-methyl-*N*-vanylyl-6-nonenamide, C_18_H_27_NO_3_) is the most pungent and abundant, accounting for about 70% of all capsaicinoids, followed by dihydrocapsaicin (~22%), nordihydrocapsaicin (~7%) [2]. The vanilloid ring has a high affinity for the Transient receptor potential vanilloid subtype 1 (TRPV1), a non-selective cationic channel widely distributed in different tissues. It is highly expressed in neural (peripheral and central) tissues, especially in C fibers, and, to a lesser extent, in the Aδ fibers of the nociceptive sensory pathway [3]. TRPV1 can also be found in other tissues, including adipose tissue [4], hepatocytes [5], immune system [6], and endothelial cells [7]. As a polymodal receptor, TRPV1 responds to a broad spectrum of physical and chemical stimuli such as heat, protons, and toxins. However, it exhibits affinity (in sub-μM order), sensitivity, and selectivity for capsaicin, its primary exogenous ligand [8]. A better understanding of the TRPV1–capsaicin interaction at molecular levels has guided pharmaceutical efforts towards the use of capsaicin in the treatment of pain, as well as helping to explore new clinical uses [9]. The biological activities of capsaicin include analgesic, anesthetic [10], anti-inflammatory [11], antioxidant [12], and thermogenic effects [13]. These activities are related to capsaicin’s effects in reducing adiposity, blood pressure, blood glucose, and cholesterol levels [13,14]. Due to its pleiotropic action and high skin absorption, there is a growing interest in using capsaicin as a therapeutic alternative. Creams and patches with concentrations between 0.025–8% of capsaicin are commercially available for muscle or neuralgic pain management.

## 2. Metabolism and Bioactivity

Since capsaicin is a food compound, the natural route of capsaicin absorption is the oral via. However, its pungency limits the quantity of capsaicin administration, either in natura (in food) or as powder and capsules. Moreover, lipophilicity directly influences the activation kinetics of its receptor TRPV1 and, consequently, its ability to generate action potentials in excitable cells [15]. Thus, overcoming the capsaicin pungency maintaining its properties becomes a challenge. In this sense, capsinoids or non-pungent synthetic analogs of capsaicin, such as olvanil and arvanil (that bind efficiently to TRPV1), could replace capsaicin in some effects [16,17].

**Figure 1:**
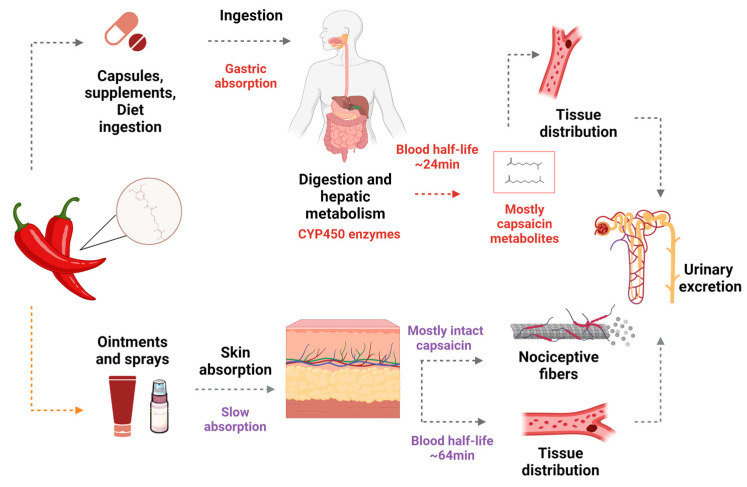
General metabolism of capsaicin orally or topically administered. Dietary capsaicin absorbed in the stomach reaches the hepatocytes via the portal system, where it is metabolized by the cytochrome P450 (CYP450) enzyme system. When given through topical administration, capsaicin is rapidly absorbed and undergoes slow biotransformation, mainly remaining intact, without hepatic biotransformation. After reaching systemic circulation, capsaicin and its metabolites are distributed to peripherical organs and trigger systemic effects. Finally, they are eliminated mainly through the kidneys. Created with BioRender.com.

After oral ingestion, 50–90% of capsaicin is passively absorbed in the stomach, and its maximum concentration in blood is seen 1 h later [18] (Figure 1). First-pass metabolism occurs in the liver, mediated by the cytochrome P450 system, which generates metabolites such as 16-hydroxycapsaicin, 17-hydroxycapsaicin, nordihydrocapsaicin, 16,17-dehydrocapsaicin, vanillin, and vanilamine. In systemic circulation, capsaicin and its derived compounds are transported, linked to albumin, and distributed to organs and tissues [2].

When applied topically to the skin, capsaicin is rapidly absorbed and undergoes slow biotransformation, remaining primarily intact, with only a small part biotransformed into vanillin and vanillic acid. Unlike oral intake, topical applications prevent the hepatic metabolization of capsaicin, ensuring a greater bioavailability in target tissues [19]. The half-life of capsaicin by topical is 1 to 24 h, longer than oral intake, and its excretion is mainly by the renal according to [2,19].

After topical application, the absorbed capsaicin activates its receptor TRPV1, which causes the rapid influx of sodium ions (Na^+^) and calcium (Ca^2+^) from the extracellular environment to the cell interior. Then, a depolarization cascade is initiated and transmitted along sensory fibers from the spinal cord to the brain, causing pain sensation. A long-term refractory state follows the initial pain sensation, and the sensory neurons stop responding to an additional application of capsaicin in a process called “desensitization” [20,21] (Figure 1).

## 3. TRPV1-Dependent Mechanisms of Action

Capsaicin binds to the transmembrane segments of TRPV1 channels in a “tail up, head down” configuration and initiates calcium influx and desensitization of nerve fibers [8]. In the case of chronic use of capsaicin, during the first few applications, it promotes the release of the preformed substance P, a potent local pain signal, which causes an initial state of neurogenic inflammation. The depletion of substance P and inoperability of nociceptive cells due to the constant influx of calcium occur after repeated applications, which prevents the formation of more mediators and reduces the inflammatory state [22]. In summary, to minimize pain, capsaicin first causes hyperalgesia, which, by exhaustion, leads to desensitization and a state of analgesia [23].

The rapid ionic influx of Ca^2+^ caused by the TRPV1 activation generates activation of second messengers and modulation of several metabolic pathways. TRPV1 is also expressed in non-excitable cells, such as adipose tissue [4]. In this tissue, capsaicin can exert anti-obesogenic and thermoregulatory effects via TRPV1 activation [24] by increasing thermogenic gene expression such as uncoupling protein 1 (UCP-1), Sirtuin 1 (SIRT-1) [25] and peroxisome proliferator-activated receptor -γ (PPARγ) coactivator 1α (PGC-1α) [26,27]. These factors can interfere with lipid metabolism by suppressing inflammatory responses, increasing lipid oxidation, inhibiting adipogenesis, activating brown adipose tissue, and increasing satiety by interfering in the hypothalamic neuronal circuits [27,28].

**Figure 2:**
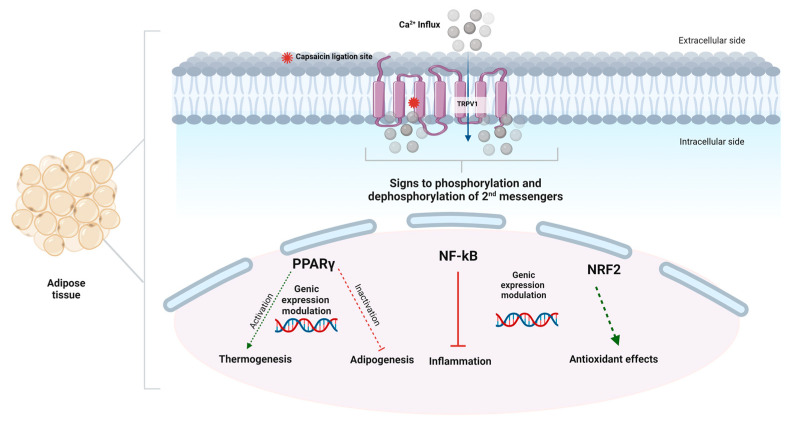
General scheme of the main effects described for the TRPV1-dependent action of capsaicin. The rapid influx of calcium caused by the binding of the capsaicin molecule to its receptor triggers a cascade of signals that include phosphorylation and dephosphorylation of second messengers and enzymes, regulating mainly the pathways of nociception (in neural tissue), thermogenesis, oxidative stress, inflammation, and adipogenesis in adipose tissue. Created with BioRender.com.

The higher intracellular calcium concentration promoted by the Capsaicin-TRPV1 binding may increase the expression and activity of the endothelium-specific transcription factor (KLF2), which could increase the expression of the enzyme endothelial nitric oxide synthase (eNOS) and, consequently, the availability of nitric oxide (NO) (Figure 2). Moreover, capsaicin also induces expression [29] while decreasing the expression of inflammatory biomarkers such as IL-6, TNF, and CCL-2, associated with NF-κB inactivation [30]. Phosphorylation of Akt is also described after capsaicin treatment, which results in disruption of the NRF2/Keap complex and release of activated transcription factor NRF2 (Figure 2). This signaling triggers the transcription of heme-oxygenase1 genes, which are essential for heme degradation and prevention of oxidative damage [31]. Other antioxidant enzymes, such as superoxide dismutase (SOD), catalase, and glutathione peroxidase, as well as glutathione levels, may have their activities modified after oral treatment with capsaicin [32]. Figure 2 summarizes the main TRPV1-dependent effects of capsaicin.

## 4. TRPV1-Independent Mechanisms of Action

**Figure 3:**
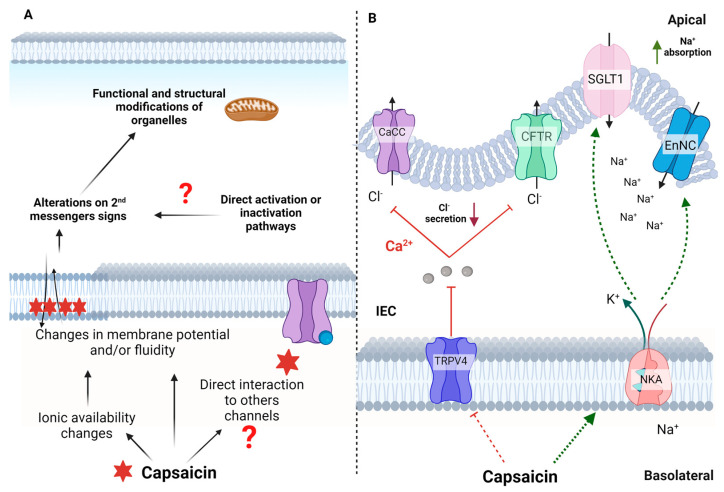
Main actions carried out by capsaicin independently of TRPV1. (**A**) Regardless of the presence of receptors, capsaicin could interact with biological membranes and modify membrane fluidity or potential, triggering signals conducted by the involved second messengers, which causes structural changes in cells and organelles, including mitochondria. These structural changes culminate in physiological responses in non-neuronal tissues that may be related to capsaicin’s beneficial or adverse actions. Furthermore, it is suggested that capsaicin could also directly bind to receptors or channels other than TRPV1 and generate changes in the metabolic pathways related to these receptors. (**B**) In intestinal epithelial cells (IECs), capsaicin could induce changes in other ion channels, such as TRPV4, blocking their actions. This blockade attenuates Cl^−^ secretion and stimulates Na^+^ absorption. NKA (Na/K ATPase); (ENaC) epithelial Na^+^ channels; (CFTR) cystic fibrosis transmembrane conductance regulator; (SGLT1) Na^+^-glucose cotransporter 1. Created with BioRender.com.

Although TRPV1 is the central mediator of capsaicin’s effects, studies suggest this receptor is not its only target (Figure 3). The activations of TRPV1-dependent or independent pathways by capsaicin are related to the dose of capsaicin and the site where capsaicin is acting [21]. For example, in the gastrointestinal tract, low concentrations of capsaicin (~1 μM) are reported to induce cell death by mechanisms involving a rapid and transient increase in TRPV1-dependent intracellular Ca^2+^. However, higher concentrations of capsaicin (≥10 μM) induce cell death through TRPV1-independent mechanisms involving mitochondrial dysfunction and plasma membrane depolarization [27].

Despite extensive research, the exact mechanisms of non-neuronal and TRPV1-independent effects of capsaicin are poorly understood. A non-specific and receptor-independent effect, such as the induction of changes in membrane fluidity, is also dependent on membrane lipid composition [33]. Capsaicin can cause structural and physiological changes in organelles, such as mitochondria, by chemically affecting the structural properties of biological membranes. These changes occur because the physicochemical modifications in this nanoenvironment can alter the membrane potential and the ionic concentration and cause oxidative imbalance. These effects could culminate in apoptosis, as reported in pancreatic cancer cell lines [32].

The direct effects of capsaicin seem to be affected by the cell type, in addition to the dose and route of administration. Capsaicin acts not only through increases in Ca^2+^ mediated by its binding to TRPV1 but also by modifying the availability and flux of other ions such as Na^+^, K^+^, and Ca^2+^ itself for other voltage-gated ion channels, ionic transporters, and possibly interacting directly with other channels and receptors [34].

It is suggested that capsaicin induces impaired hippocampal *Gamma oscillations* through a TRPV1-independent pathway involving Na^+^/K^+^ ATPase (NKA). Thus, using capsaicin to reduce these oscillations to levels of healthy controls could become a promising strategy in the face of psychiatric and neurological disorders that present this dysfunction as a characteristic [21].

Another possible TRPV1-independent effect of capsaicin is its oncoprotective action. In glioblastoma and colon cancer cells, capsaicin was able to induce apoptosis by increasing PPARγ expression independently of its vanilloid receptor [35]. Some of the TRPV1-independent effects of capsaicin may be related to its ability to activate or deactivate PPARs, particularly PPARγ [36]. PPARγ is predominantly expressed in adipose tissue and, to a lesser extent, in other tissues such as skeletal muscle and liver. Capsaicin is described as a PPARγ activator or inhibitor. This effect in modulating PPARγ seems to be dependent on several factors, such as the balance between the PPARs (which also include PPARα and PPARβ/δ) in the different metabolic states, the presence of TRPV1, the dose of capsaicin, and even the intestinal enterotype [37,38]. Therefore, the possibility of directing the actions of capsaicin in PPARγ for therapeutic purposes and, at the same time, minimizing side effects represent major challenges [39].

Dietary capsaicin can attenuate Cl^−^ secretion and stimulate Na^+^ absorption by blocking TRPV4 channels while stimulating NKA activity in intestinal epithelial cells (IEC). The intracellular Ca^2+^ stimulates Cl^-^ secretion through the Ca^2+^-dependent Cl^−^ secretion (CaCC) channel and apical cystic fibrosis transmembrane conductance regulator (CFTR). The blockade of TRPV4 caused by capsaicin decreases Ca^2+^ entry through this channel and reduces the stimulus for Cl^−^ secretion. In contrast, stimulation of basolateral NKA activity would establish an ionic gradient and driving force to promote apical Na^+^ absorption via Na^+^-glucose transporter 1 (SGLT1) and the epithelial apical Na^+^ channel (ENaC) [40].

It is suggested that the protective effects of capsaicin against chemical carcinogens are mainly related to halogens metabolized by the cytochrome P450 (CYP450) enzyme system. Several mechanisms make capsaicin a promising agent against several types of cancer, as highlighted in a recent review [41]. Capsaicin acts by inhibiting the activity of these enzymes, which prevents the metabolization of halogens into highly reactive species. Thus, the chemoprotective role of capsaicin has been mainly associated with the ability to modulate CYP enzymes [42].

Recent approaches are centered on capsaicin’s ability to modulate the intestinal microbiota. Capsaicin can influence intestinal microbiota by a mechanism not totally understood. It is suggested that regular treatment with capsaicin increases diversity in the gut microbiota and abundance of short-chain fatty acid (SCFA)-producing bacteria [43].

Moreover, capsaicin treatment prevents dysbiosis, gut barrier dysfunction, and low-grade chronic systemic inflammation [44] caused by dysbiosis. The ability of capsaicin to modulate the intestinal microbiota has been discussed not only as one of its TRPV1-independent effects but also as the basis for other beneficial systemic effects [45] on metabolic diseases [46] and cancer [47]. Nonetheless, although this is a promising field of research, the role of capsaicin on intestinal microbiota is still controversial [48]. Such studies were carried out primarily in experimental models; despite their promising effects, caution is needed when translating such effects into humans.

## 5. Anti-Inflammatory and Gastrointestinal Effects of Capsaicin

Capsaicin has been tested for preventing and treating intestinal pain and inflammation due to its functions in the gastrointestinal tract [49,50,51]. Its actions on gastrointestinal sensory neurons may be dependent or independent of TRPV1 in mammals. Capsaicin triggers a painful and burning sensation in a TRPV1-dependent manner. On the other hand, as cited before, capsaicin appears to induce changes in anion secretions and induction of apoptosis of cancer cells by mechanisms independent of TRPV1 [28,52]. However, little is known about the actions of capsaicin in the gastrointestinal tract independently of TRPV1 [40]. Recent evidence has shown an important role for TRPV4 channels in the pathogenesis of experimental ulcerative colitis because these channels regulate ion transport in the intestinal epithelium [40]. Capsaicin was able to inhibit Cl^−^ secretion and promote Na^+^ absorption by blocking TRPV4 channels. Moreover, the inactivation of TRPV4 channels by capsaicin in experimental colitis suppressed the overactivation of these channels that occurs in colitis [40].

Regarding anti-inflammatory effects, one of the main actions of capsaicin is the inhibition of the NFκ-B pathway that reduces proinflammatory cytokines (IL-6, TNF, and IL-1β) production and mRNA expression of the NLRP3 inflammasome, associated with a lower NFκB phosphorylation [53]. In addition, the reduction in the junctional protein E-cadherin, typical in *H. pylori* infection, was attenuated after capsaicin treatment. It occurred due to reduced expression of the gastric cancer biomarkers miR21 and miR223, which downregulate the E-cadherin expression [53].

However, the effects of capsaicin on inflammation can be modulated by the dose administered. Xiang et al. [54] investigated the effects of oral administration of 40, 60, and 80 mg/kg weight of capsaicin for 7 days on gastrointestinal health and intestinal microbiota, using specific pathogen-free (SPF) mice. They showed that intakes above 60 mg/kg seem harmful to the intestine. The administration of 80 mg/kg caused inflammatory cell infiltration and loss of mucus-producing goblet cells in the colon. Such inflammation was characterized by elevated levels of inflammatory cytokines, especially TNF and IL-β1, and lower levels of the anti-inflammatory cytokine IL-10, suggesting damage to the jejunum and colon at such a dose. In addition, there was an increase in serum levels of neuropeptides (SP and CGRP) related to visceral pain, causing additional damage to the gastrointestinal system in a TRPV1-dependent manner [54]. On the other hand, changes in the intestinal microbiota profile were observed when 40 mg/kg was assessed. The abundance of *Lactobacillus* in the jejunum and ileum associated with capsaicin treatment was related to higher levels of IL-10, suggesting that the microbiota modulation by capsaicin may induce changes in the intestinal inflammatory environment.

In an experimental model of colorectal cancer, Cheng et al. [48] administered 300 mg/kg of capsaicin for 12 weeks to mice before the intraperitoneal injection of EGFP-labelled CT26 cells. Mice presented a proinflammatory microenvironment with increased cytokines IL-12, TNF, and IFN in the liver and IL-6 in the blood. In addition, there was an increase in neutrophils, macrophages, and monocyte infiltration associated with a reduction in the thickness of the intestinal mucus barrier, bacterial translocation, and destruction of the vascular epithelial barrier. The authors suggested that intake of high-dose CAP over the long term could increase the risk of metastasis in colorectal cancer [48]. However, the results contradict other studies indicating capsaicin could suppress tumor cell growth [54,55,56]. As discussed above, some effects of capsaicin are dose-dependent and may differ according to its concentration and time of use. For instance, there is no reason to recommend or avoid using capsaicin-rich foods in those with intestinal diseases [27,48,55,56,57].

## 6. Capsaicin and Non-Communicable Chronic Diseases

Capsaicin has been suggested as a potential alternative treatment for diabetes due to its influence on glucose metabolism [32]. The in vitro study of Bort et al. [36] using C1C12-derived myotubes cultured with 100 and 200 μM of capsaicin found an increase in intracellular calcium induced by CAP-TRPV1 binding. It could stimulate intracellular signaling pathways through calcium/calmodulin-dependent protein kinase kinase 2 (CAMKK2), which, in turn, phosphorylates AMP-activated protein kinase (AMPK), a regulatory kinase involved in glucose and lipid metabolisms. This action would improve insulin sensitivity due to increased fatty acid oxidation, insulin secretion, glucose uptake, and reduction in inflammatory factors [36].

Capsaicin was also related to increased glucose uptake in a TRPV1-independent, TRPV4-dependent manner, stimulating the co-transport of Na-glucose. In TRPV4 knockout mice, Na^+^ uptake was increased compared to the wild type, in which CAP was also shown to be able to potentiate the action of blocking TRPV4 and Na absorption via the Na-glucose cotransporter (SGLT1) [40,58].

In diabetic rats, oral administration of 0.5 g/kg of capsaicin for 8 weeks reduced cholesterolemia, triglyceridemia, and cardiac fibrosis [59]. An analysis of the heart showed increased expression of TRPV1 and eNOS associated with increased levels of nitric oxide and reduced reactive oxygen species (ROS). Similar results were obtained when vascular endothelial cells from these animals were treated with different concentrations of glucose and capsaicin [59].

Individuals with chronic diseases such as diabetes are more prone to vascular senescence, manifested by accelerated vascular stiffness and atherosclerosis. Senescent endothelial cells show loss of proliferation potential, decreased vasodilation, and proinflammatory and pro-arteriosclerotic phenotype [60]. In this sense, the beneficial effects of capsaicin at different concentrations (0.3 µM, 1.0 µM, and 3.0 µM) on the senescence of HUVEC cells exposed to 5 and 33 mM of glucose were studied. The findings showed a capsaicin-induced increase in sirtuin 1 (SIRT1), a protein that prevents endothelial senescence induced by intermittent hyperglycemia. In addition to increasing SIRT1 levels, capsaicin was able to suppress p21, a protein associated with ROS production and senescence. Thus, it was suggested that capsaicin could be a potential adjuvant in treating vascular aging in diabetes [60].

Reports of the action of capsaicin in atherosclerosis development are numerous, mainly due to the improvement in lipid profile, inflammation, and endothelial dysfunction [46,60,61,62,63]. Dai et al. [46] studied the role of the microbiota in the anti-atherosclerotic effect of oral capsaicin (0.01% *w*/*w*) in ApoE knockout mice fed a high-fat diet (HFD) and demonstrated that the reduction in LDL-c and increase in HDL-c was associated with reductions in atherogenesis shown by the reduction in the lesion area on aortic sinus and greater stability of atherosclerotic plaques. In the context of inflammation, capsaicin improved the HFD-induced inflammatory response in the intestinal mucosa, as seen by the reduction in inflammatory biomarkers IL-6 and LPS. In addition, capsaicin administration also altered the composition of the intestinal microbiota and metabolomic profiles, which could contribute to improving atherosclerosis. The microbiota’s relevant role in capsaicin’s action was confirmed when these effects disappeared in antibiotic-treated animals [46].

Regarding experimental studies on body weight control, Song et al. [64] showed that diets with low (0.01%) or high (0.02%) capsaicin supplementation could not prevent weight gain in ob/ob mice but inhibited increased fasting blood glucose and improved insulin sensitivity. Despite the absence of effects on body weight, capsaicin, in both concentrations, increased fecal levels of butyrate (short-chain fatty acid produced by microbiota fermentation) and blood GLP-1 while decreasing blood levels of ghrelin and inflammatory cytokines [64]. Although both levels of supplementation failed to alter the α and β diversity of the intestinal microbiota, there was an increase in the *Firmicutes*/*Bacteroidetes* fila ratio. At the genus level, an increase in the abundance of Roseburia was found, related to lower blood glucose levels, and a decrease in the abundance of *Bacteroides* and *Parabacteroides* associated with higher blood glucose [64].

Together, these data corroborate previous studies showing the influence of the microbiota-modulating action in the hypoglycemic and anti-inflammatory effects of Capsaicin [27,48,49,65]. Nonetheless, although these preclinical studies show encouraging results, they must be confirmed through long-lasting controlled trials.

## 7. Effect of Capsaicin on Metabolic Syndrome and Obesity

Many studies with capsaicin have been performed in rodents, and the resulting transposition to humans is not always possible, including dose matching for humans. In this way, Szallasi et al. [66] estimated the correspondence of dietary capsaicin intake from rats to humans. They concluded that a dose of 50 mg/kg of supplemented capsaicin for rats (about 250 g of body weight) would be equivalent to 12.5 mg/day for humans, an intake higher than the average consumption in Korea, estimated in 2.17 mg of Capsaicin [67]. Furthermore, the 0.01% capsaicin supplementation in diet, used in several experimental models, is also above the average intake in most countries that consume spicy meals. Thus, studies on realistic amounts of capsaicin supplementation or foods rich in capsaicinoids should be conducted. Moreover, these studies should consider individual differences, such as the presence or absence of obesity and its metabolic consequences.

The described beneficial properties of capsaicin occur due to a wide variety of properties, which goes beyond the metabolism and expands its field of use from packaging conservation to effects on human health [68,69].

The metabolic actions of capsaicin, mainly related to antioxidant, anti-inflammatory, and thermogenic properties, make capsaicin a promising adjuvant in treating and preventing metabolic syndrome, obesity, and associated comorbidities such as dyslipidemia and diabetes mellitus [69,70,71,72].

A previous scope review analyzed publications about capsaicin receptors signaling for its antiobesity properties [73]. The analysis showed that, besides TRPV1, capsaicin could also act as an agonist for PPARs, especially PPARγ, on fat metabolism [36,74]. Given the role of PPARγ in the transcriptional regulation of adipogenesis, this would be one of the potential pathways for the antiobesity action of Caps. Activation of PPARγ by capsaicin has also been associated with its beneficial action on obesity, reducing inflammatory markers and inducing the expression and synthesis of adiponectin in mouse adipocytes [73].

Thus, capsaicin signaling in obesity and fat metabolism is mainly described as TRPV1-dependent [25,75,76,77,78], although some studies presented TRPV1-independent actions, including activation of PPARγ [4,76,79,80]. In fact, independently of capsaicin signaling, several mechanisms mediate the action of capsaicin in weight loss, mostly linked to its ability to increase thermogenesis, induce satiety, and modify the obesogenic microbiota.

Although several reviews have been published in recent years about the effect of capsaicin, capsinoids, and spicy foods on obesity [24,41,66,81,82], we are far from the confirmatory answer to if this action occurs in the general as well as in the affected population, what the magnitude of this action is, if it is safe, and what the optimal therapeutic dose and treatment duration are.

### 7.1. Effects of Capsaicin on Food Intake and Satiety

Capsaicin, through its binding to albumin, reaches the adrenal gland, which induces the release of catecholamines. It has been shown that capsaicin can increase lipolysis in adipocytes and stimulate fat oxidation by activating the sympathetic nervous system [83]. This situation may affect tissues such as brown and white adipose tissue involved in body weight control. In white adipose tissue, capsaicin can exert several actions, increasing lipolysis and reducing lipogenesis [84,85] and, thus, reducing adipocyte volume and, consequently, adiposity [86]. In brown adipose tissue, capsaicin activates browning by increasing UCP1 expression, mitochondrial biogenesis, energy expenditure, and glycerol recycling [85,87,88].

Studies in animal models have suggested that capsaicin may increase satietogenic hormones such as glucagon-like protein 1 (GLP-1) and the gastric inhibitory peptide (GIP) [89]. The intense concentration of TRPV1 in the nervous system, mainly in the hypothalamus, supports the possible relationship between capsaicin and changes in hunger/satiety control. However, the role of capsaicin in increasing these incretins in clinical studies is still inconsistent. In this way, some reviews assessed the effect of capsaicin and spicy food on cognition, food preferences, and satiety induction [24,81,90,91,92]. Although an increase in GLP-1 was seen after a capsaicin-supplemented meal [93], a more recent study using intraduodenal infusion of capsaicin in volunteers without obesity did not observe an increase in plasma concentrations of GLP-1 and PYY [94].

Rigamonti et al. [95] assessed the acute effect of capsaicin on appetite-regulating gastrointestinal peptides on the energy balance of young subjects with obesity. They showed that capsaicin (2 mg), provided during an “ad libitum” dinner, did not change circulating levels of ghrelin, GLP-1, peptide YY, or satiety after food. Nonetheless, the pre-post meal difference in rest metabolism was higher in those receiving capsaicin. The authors suggest capsaicin could act as a metabolic activator rather than a hypophagic inducer [95]. In another study, supplementation for 8 weeks with 12 mg/day of capsiate, a non-pungent analog of capsaicin, increased body weight by 1 kg. However, this increase was due to increased upper body strength compared to placebo [96].

It is interesting to note that the effect of capsaicin may change between individuals, depending on body weight. It was the result of a meta-analysis that found that after ingestion of capsaicin or capsinoids, there is a modest increase in energy expenditure of around 58 kcal/day and a reduction in the respiratory quotient, suggesting increased lipid oxidation in individuals with BMI > 25 kg/m^2^ [97]. In those with BMI < 25 kg/m^2^, capsaicin or capsinoids did not affect energy expenditure or respiratory quotient. In this way, research addressing the long-term effect of capsaicin in individuals with and without obesity should be conducted, analyzing the effects on food intake, satiety, and intestinal hormone production. The diet composition should also be considered since it affects hormone release and central food control.

### 7.2. Effect of Capsaicin on Obesogenic Dysbiosis

The effects of capsaicin in favoring healthy gut microbiota have been demonstrated in experimental animals [98,99,100,101] and in vitro studies [43,102], with a lag in the literature regarding clinical studies. One factor in chronic low-grade inflammation linked to obesity is metabolic endotoxemia, resulting in intestinal dysbiosis. Kang et al. [103] demonstrated that mice fed a high-fat diet supplemented with capsaicin exhibited lower levels of metabolic endotoxemia and chronic inflammation associated with lower body weight gain. Other findings in the gastrointestinal tract were increased abundance of butyrate-producing *Ruminococcaceae* and *Lachnospiraceae*, and low levels of S24-7 lipopolysaccharide (LPS)-producing bacteria and inhibition of type 1 cannabinoid receptors (CB1). Interestingly, when the microbiota from the capsaicin-treated animals was transferred to germ-free mice fed a high-fat diet, the recipient mice maintained the protective effects.

Furthermore, when microbiota depletion was induced by an antibiotic cocktail, there was a loss of capsaicin protection against obesity [103]. However, Manca et al. [104], in an exploratory clinical study with women presenting overweight and obesity, showed that the oral administration of capsicum extract capsules for 12 weeks produced a modest increase in the relative abundance of the genus *Flavonifractor*. The rise in the *Flavonifractor* genus may be related to its ability to metabolize flavonoids such as curcumin, with which capsaicin has structural homology. Species belonging to this genus, such as *F. plautii*, have been involved in reducing inflammation [105].

**Figure 4:**
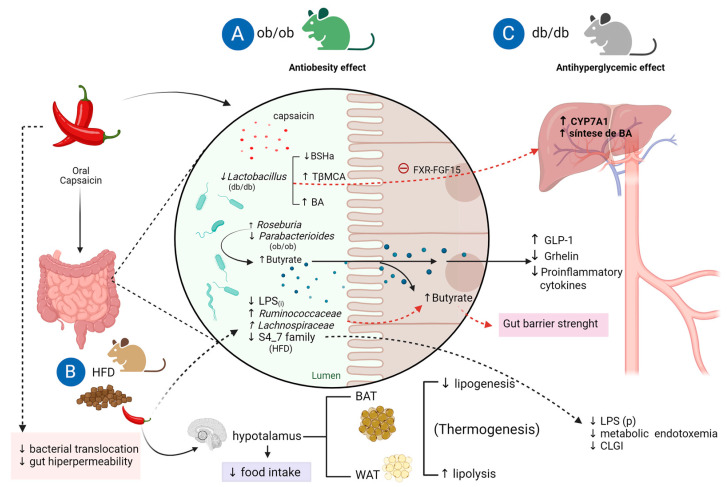
Influence of dietary capsaicin on glucose homeostasis and obesity via modulation of the gut microbiota. (**A**). capsaicin increases the abundance of Roseburia. It decreases the abundance of Bacteroides and Parabacterioides in obese diabetic mice (ob/ob), leading to an increase in butyrate and GLP-1 and a decrease in total plasma ghrelin and also and proinflammatory cytokines. (**B**). The antiobesity effects of capsaicin in mice fed a high-fat diet (HFD) modulate the gut–brain (hypothalamus) axis by decreasing food intake. In addition, capsaicin induces browning, favoring the formation of brown adipose tissue (BAT) and stimulating lipolysis of white adipose tissue (WAT). Capsaicin reduces the abundance of Gram-negative bacteria that secrete intestinal lipopolysaccharide (LPS(i)) and increases the abundance of butyrogenic bacteria (Ruminococcacea and Lachnospiraceae) and, consequently, butyrate. This effect attenuates the increased intestinal permeability and bacterial translocation caused by HFD and suppresses cannabinoid intestinal receptor type 1 (CB1(i)) expression. Thus, dietary capsaicin strengthens the intestinal barrier due to the increase in butyrate production, decrease in LPS(i) and plasma LPS (LPS(p)) with reduction in metabolic endotoxemia and chronic low-grade inflammation (CLGI) (**C**). The antihyperglycemic effect of capsaicin is due to a decrease in the abundance of Lactobacillus in diabetic mice (db/db), causing a reduction in the activity of bile salt hydrolase (BSHa) and an increase in the levels of conjugated bile acids (BA) in the intestine and of β-muricholic tauro acid (TβMCA), a farsenoid receptor antagonist (FXR), leading to the suppression of the FXR-FGF15 axis and a positive regulation of the expression of cholesterol 7 hydroxylase (CYP7A 1) stimulating the hepatic synthesis of BA. Created with BioRender.com.

Xia et al. [59] cultivated human fecal microbiota from healthy voluntaries with red pepper (2%) for 24 h and found an increase in acetate and propionate concentrations compared with control cultures. *Subdoligranulum* spp.-, *Blautia* spp.-, *Faecalibacterium prausnitzii*-, *P. vulgatus*-, and *Prevotella copri*-like bacteria were defined as red pepper-responsive indigenous gut bacteria. The authors concluded that red pepper increases the short-chain fatty acid-producing bacteria and other beneficial bacteria in human fecal cultures. A similar result was seen in the study of Mahalak et al. [43], showing that capsaicin increases the diversity of human microbiota and SCFA abundance. These studies could explain the beneficial health effects of capsaicin in obesity and other non-communicable chronic diseases. The influence of capsaicin on glucose homeostasis and obesity by modulation of the gut microbiota is summarized in Figure 4.

In conclusion, clinical studies exploring the role of intestinal microbiota on the capsaicin effect, as well as the effect of capsaicin on the microbiome and intestinal microbiota balance, are necessary to assess the impact of this interaction on several metabolic conditions.

### 7.3. Effect of Non-Pungent Capsinoids and Spice Foods

Capsiate, dihydrocapsiate, and nordihydrocapsiate belong to the family capsinoids with a molecular structure similar to capsaicin. Like capsaicin, capsinoids bind TRPV1 in the intestinal epithelium. However, they do not activate TRPV1 in the oral cavity. Hereafter, they can induce their biological effects without producing pungency. In this way, capsinoids such as capsiate could be, as occurs with capsaicin, a promising agent for treating obesity.

Gupta et al. [71], in a review of the benefits of capsiate, concluded that it shares several properties with capsaicin, including increased satiety and reduced body weight. Likewise, Zsiborás et al. [97], in their meta-analysis, concluded that the metabolic effects not only of capsaicin but also of capsiate are significant in individuals with high BMI. However, in the same year, Szallasi et al. [66], analyzing clinical studies carried out with capsiate, did not observe a significant influence of non-pungent capsinoids on body weight and obesity.

Some reviews and clinical studies carried out with red [71,84,106] or black pepper [107,108] have observed beneficial effects on human health. Since capsaicin and chili peppers are the main components that give spicy characteristics to foods, it could be expected to associate the increase in spicy food consumption with the increase in capsaicin’s beneficial effects. In this way, Yang et al. [109] examined the association between the frequency of spicy food intake and abdominal obesity in a Chinese population of 40,877 individuals. Interestingly, the results showed that the higher consumption of spicy foods was associated with increased abdominal adiposity. However, other factors besides capsaicin content, such as total caloric and nutrient intakes, physical activity, and comorbidities, among others, could have contributed to this result.

Similar results were found in a meta-analysis to evaluate the association between consumption of spicy foods and overweight/obesity, high blood pressure, and blood lipid profile [67]. The results showed that greater consumption of spicy foods was associated with an increased risk of overweight/obesity, an increased LDL-c, and a modest reduction in HDL-c. However, the consumption of spicy foods was negatively correlated with hypertension. Another study [110] analyzed the profile of spicy food consumption in a Chinese population. The results showed that the more frequent consumption and intense pungency of spicy foods were positively correlated with a preference for deep-fried food, salty snakes, alcohol and tea drinking, and tobacco smoking. These results highlight the importance of distinguishing between isolated capsaicin and pepper-containing foods’ effects.

In addition to capsaicinoids and capsinoids, peppers are sources of nutrients and other bioactive components such as flavonoids, phenolic compounds, carotenoids, and ascorbic acid. These compounds aggregate and enhance several properties in peppers beyond capsaicin effects [92,111]. Other issues, such as the cultural consumption of pepper and the genetic characteristics of the studied populations, can influence the results. In this sense, clinical studies in populations from countries with the highest levels of per capita pepper consumption, such as Bulgaria (21 mg/day), Singapore (14 mg/day), and Vietnam (0.4 mg/day), as well as studies that consider different genetic characteristics, such as the presence of polymorphisms or different proteoforms of vanilloid receptors, can provide important findings and a better understanding of the role of capsaicin in health and diseases.

We can conclude that using capsinoids and pepper to improve chronic diseases is still controversial, mainly considering spicy foods in epidemiological studies. In this way, it is not possible to recommend or avoid spicy foods for such diseases.

## 8. Adverse Effects

Capsaicin is not free from adverse effects, especially when given topically. The most common effects, considering all routes of administration, are burning sensation, pruritus, edema at the administration site, and pain [112]. Muscle aches, chills, nasopharyngitis, sinusitis, bronchitis, cough and dyspnea, fever, tachycardia, dizziness and headache, hypertension, nausea, and vomiting are also described, although less common. Rarer complications include dysgeusia, hypoesthesia, peripheral edema, peripheral sensory neuropathy, and throat irritation [112]. Nonetheless, most effects may disappear during treatment.

## 9. Conclusions

Studies using capsaicinoids and capsinoids as adjuvants in obesity, diabetes, dyslipidemia, or cancer therapy are increasing in the literature. Although capsaicin is already approved as a topical treatment for neuropathic pain, its use in non-communicable diseases has not been properly and extensively studied to permit us an incontestable conclusion. Most of these studies show beneficial properties, improving antioxidant and anti-inflammatory status, inducing thermogenesis, and reducing white adipose tissue. Other mechanisms are also described, including reducing food intake and improving intestinal dysbiosis. Although some clinical studies lead us to the benefits of capsaicin supplementation, few studies were performed over 12 weeks in a population affected by these diseases. We should also further explore the route of administration for each disease since the metabolism of capsaicin depends on its route of administration.

Regarding obesity, several reviews have been published in recent years on the effect of capsaicin, capsinoids, and spicy foods on weight loss, making the indication as an adjuvant in this disease likely in the medium term. Type 2 diabetes and dyslipidemia are closely linked to obesity and, consequently, could be beneficiated by capsaicin supplementation. However, at the moment, there is still a lack of information about the following: 1—whether capsaicin action is similar in the general population and those affected by the disease; 2—the optimal therapeutic dose; 3—the duration of treatment; and 4—the safety of long-term administration. Even less is known about the effects of capsaicin on the gut microbiota and how this could reflect on metabolic diseases. In this way, many studies are still needed to characterize its action on dysbiosis. Regarding the role of capsaicin in cancer prevention, we are only in the first steps. Much remains to be researched, including understanding whether capsaicin is a cancer-protective or -promoting agent.

Furthermore, non-pungent capsinoids or synthetic analogs can conveniently substitute capsaicin. However, meta-analyses, although mostly showing beneficial effects in obesity, diabetes, dyslipidemia, and cancer, still need confirmation from new clinical trials.

Therefore, the answer to the question “Are we ready to recommend capsaicin for disorders other than neuropathic pain?” is no, we are not. We are close, but not ready yet, to including capsaicin supplements or creams as an adjunct in treating obesity and type 2 diabetes. Furthermore, we are far from fully understanding the role of capsaicin in other non-communicable diseases.
